# A New Method of Making Anatomical Preparations

**Published:** 1834-01-01

**Authors:** 


*A New Method of making Anatomical Preparations; particularly those relating to the Nervous System.* By JOSEPH SWAN. *Third Edition, considerably Enlarged.*—London, 1833. 8vo. pp. 111.

THERE are books to which the mere name of the author is a sufficient passport, and such is the one which is now before us. A work on anatomical preparations from Mr. Swan is so much honey from Hybla; an excellent thing, from the best possible source. The advantage to the learner of preserving the result of his dissections for future reference is so great, especially if he intends to practise in the country, that it is needless to say much upon the subject; and we shall therefore content ourselves with recommending every student of anatomy to make preparations, and every one who makes preparations to buy Mr. Swan's book.

Some superficial persons may think the subject dry, but to us there is a great deal of spirit in our author's manner of treating it. The following extract will give our readers some notion of the laudable minuteness of this excellent manual.

"*On Preparations of the Minute Nerves.* It is supposed by many that young children are better subjects for dissections of the nerves than those of more advanced age, but this is a great mistake. In a child the nerves are certainly larger in proportion to the other parts than in the adult, and the larger nerves may be conveniently enough dissected in them, but it is not so with the more minute branches.

"Even in a very thin child so much fat is always found in different parts as to make the dissection very tedious, besides, the nerves have not appeared so strong as in the adult, so that much disappointment has been occasioned by their frequent breaking.

"The minute nerves of the face and neck are generally the largest and most distinct in the male, and one should be chosen who has been originally strong and well-made, between twenty and fifty years old, and as free from fat as possible. The nerves are almost invariably found more delicate in the female than in the male, and in very advanced age so much so as to be totally unfit for dissection.

"Although it may not be in the power of the anatomist to choose such a subject as he desires, yet for a dissection of the sympathetic nerve and par vagum, one emaciated, but not destroyed by a disease of the lungs, is to be preferred; for it generally happens in cases of this sort, that, besides the disease of these viscera, all the absorbent glands and vessels of the neck and thorax are so much enlarged, and there is frequently such a thickening of the parts at the bottom of the neck, as to create a degree of confusion, which renders the dissection of the sympathetic nerve and par vagum sometimes unsatisfactory.

"What has been said about the choice of human subjects, as far as respects the age, does not apply in the same manner to animals, for in many of these, and especially the larger ones, the nerves in general are never so conveniently dissected as they are within a week or two after birth, because at that age they are generally more free from fat in every part than at any later period, as it is so difficult to procure such as have been in a diseased state long enough for all the fat to have been absorbed.

"It is better to separate most of the nerves from the fat before attempts are made to remove much of it, but in hot weather it becomes very soft, so that much of it may be absorbed by blotting paper, and then the remaining membrane that inclosed it can be separated without much difficulty, or the risk of destroying the minute branches of skin on the tip of the nose and margin of the lips.

"In dissecting the nerves of the neck, and indeed those that are minute in every other part of the body, the scalpel must be very little used, except for removing the skin; for, when the nervous branches are very numerous, it is next to impossible to separate them from the surrounding parts with an instrument of this kind, let the care and knowledge of the anatomist be ever so great. In a general way, the most convenient instrument will be one similar to a separate blade of a pair of scissors, made to cut like a knife, which may be procured at Mr. Laundy's, St. Thomas's street, Southwark.

"The student is not to cut straight forward when he is following a nerve, but he must take hold of the trunk, and separate it from the surrounding parts rather by poking and tearing away these than by cutting, although he may frequently use the cutting edge advantageously when he can see that he shall do no mischief. The instrument should generally be used by keeping the back of it to the subject, and directing the point to enter the part obliquely downwards, so that in cutting forwards he will only divide the parts lying over the nerve; these divisions should be made by frequent short cuts, for as a nerve or a branch keeps dividing into others, if a long cut be made, either some of the branches will be divided, or others communicating with them. If he attempt to follow the nerves with the cutting edge towards the subject, he will very often be disappointed by dividing the nerves and blood-vessels; but his own experience will soon convince him of the best method of using his instruments.

"If the part is to be dried, every portion of fat must be removed, for if it be not, it will sooner or later show itself in the preparation by its transudation, which softens any varnish that may have been applied, and not only renders the preparation unpleasant by its sticking to the fingers whenever it is taken hold of, but also causes it to attract every portion of dust, so as in point of fact to take very much both from its appearance and utility.

"It is farther necessary to remove every portion of cellular membrane from the nerves, that they may be as distinct as possible, otherwise, where there are many minute branches in a small space, they will have such a confused appearance as to defeat the main purposes for which the preparations are made.

"Whilst prosecuting the dissection, it is desirable that the part should be kept in spirits, if it be probable that it will be long before it can be finished; but at all events the minute nerves should be moistened from time to time with spirit of wine, or water, as otherwise, if suffered to get very dry, they will be very liable to break.

"For making a preparation of the minute nerves, and for illustration, the sympathetic should be chosen. The integuments should be removed, and such other portions as are not required; the muscles, arteries, nerves, &c., are then to be carefully and partially separated, and the subject immersed in cold water for twenty-four hours; the nerves thus become larger, stronger, and more distinct. The dissection must then be proceeded with for some days, when the parts are to be immersed in alcohol, or salt may be used first, according to some preceding directions; for if a subject be kept in water too long, the nerves become enlarged, and the cellular membrane attached to them swollen out, but, by putting it into alcohol, they are contracted again to their natural dimensions. On taking the subject out of the alcohol, the nerves dry so fast and contract so much, as to make it absolutely necessary to have the parts not under dissection covered with a wet cloth, and the subject placed in cold water for a few hours once in every four or five days. It is thus, by alternating the use of the water and alcohol, that the subject is kept in the most proper state for dissection. When the dissection is finished, about two thirds of alcohol and one of water best preserve the natural size of the nerves." (P. 29.)

